# Are the elderly more vulnerable to psychological impact of natural disaster? A population-based survey of adult survivors of the 2008 Sichuan earthquake

**DOI:** 10.1186/1471-2458-10-172

**Published:** 2010-03-30

**Authors:** Zhaobao Jia, Wenhua Tian, Weizhi Liu, Yang Cao, Jin Yan, Zhisheng Shun

**Affiliations:** 1 Faculty of Health Service, Second Military Medical University, Shanghai, China; 2 Department of Psychology, Second Military Medical University, Shanghai, China; 3 Department of Health Statistics, Second Military Medical University, Shanghai, China; 4 Centers for Disease Control and Prevention, Aba Tibetan and Qiang Autonomous Prefecture, Sichuan province, China

## Abstract

**Background:**

The association between ages and psychological impact of natural disasters has not been well characterized. A population-based study was conducted 15 months after the 2008 Sichuan earthquake to assess whether elderly survivors were more likely to develop posttraumatic stress disorder (PTSD) and general psychiatric morbidity.

**Methods:**

A population-based survey of 327 survivors (152 elders, 175 younger adults) was conducted in severely affected areas by the earthquake, using a multi-stage systematic sampling design.

**Results:**

Compared with the younger adult survivors, the elderly were more likely to have symptoms of PTSD (22.5% vs. 8.0%, p = 0.001) and general psychiatric morbidity (42.0% vs. 25.4%, p = 0.003). Risk factors, such as being elderly, having been in serious danger, having lost family members, and having felt guilt concerning one's death or injury were significantly associated with developing PTSD; being elderly, having family members or friends seriously injured, and having felt guilt concerning one's death or injury were significantly associated with developing general psychiatric morbidity. Utilization of mental health services is strongly associated with the decreased risk for developing both of the symptoms.

**Conclusion:**

Compared with the younger adults, the elderly survivors were more likely to develop PTSD and general psychiatric morbidity. More mental health services should be distributed to the elderly and groups at particular risk, to ensure their smooth mental health reconstruction after the earthquake.

## Background

Older people are among the most vulnerable populations to the direct impact of natural disasters. Previous studies have proven that physical wellbeing of the elderly is more affected than in younger adults. For instance, a rapid assessment of the health status of people in the affected areas following the Sichuan earthquake suggested that the greatest morbidity was among those over 60 years of age [1]. After Hurricane Charley, at least one older adult's medical condition had worsened in one-third of households that included one or more older adults with a preexisting medical condition [2]. The difference has been attributed to decreased sensory awareness (sight, smell, hearing, and touch), physical impairment, chronic health conditions, and socioeconomic limitations experienced by many of the elderly [3].

Previous studies also discussed the psychological impacts of natural disasters, and posttraumatic stress disorder (PTSD) has been one of the focuses [4-6]. PTSD is a severe anxiety disorder that can develop after exposure to any event which results in psychological trauma [7]. It has been found to be the most prevalent type of psychiatric morbidity after disasters, such as earthquake and tsunami [8]. Signs and symptoms of PTSD include re-experiencing original trauma(s), by means of flashbacks or nightmares; avoidance of stimuli associated with the trauma; and increased arousal, such as difficulty falling or staying asleep, anger, and hypervigilance [7].

However, conclusions about the psychological impacts of natural disasters on elder and younger adults have been equivocal. In a population-based sample of the 1998 flood victims in China, Liu et al. reported that the elderly were over twice more likely to develop symptoms of PTSD [9], Ticehurst et al. found the elderly to be more distressed than younger adults after an earthquake [10], whereas other studies suggested that the elderly were less susceptible to psychological disorders or were consistent with the symptoms of the general population [11]. For instance, in a study undertaken 18 months after the 1988 earthquake in Armenia, no difference was found on the overall severity of PTSD symptoms between the elderly and younger adults[4], and the results of an epidemiological study after Hurricane Honduran indicated that the elderly survivors were at equal risk for developing PTSD as the younger survivors [12]. The inconsistency in the outcomes has been attributed to the properties of disasters chosen for study as well as methodological differences (e.g., methods of sampling and case detection) [6]. However, socioeconomic contexts may also play an important role in modifying the outcomes. Hence, as prior studies on the issue were mostly performed in developed countries, more information from middle- and low-income settings is needed. To our knowledge, this is the first post-disaster study that has been specially designed to investigate the psychological disparities between elderly and younger adult survivors in the developing world. In addition, we introduced utilization of mental health services as an independent variable and explored its associations with the prevalence of mental health symptoms, which was seldom discussed in previous post-disaster studies.

## Methods

### Setting and design

On May 12, 2008, a powerful earthquake measuring 8.0 on the Richter Scale struck Sichuan Province, Southwest of China. More than 69,200 people were confirmed dead, more than 374,600 were seriously injured, and more than 17,900 were reported missing [13], making it one of the deadliest natural disasters in history. The earthquake spread about 100,000 square kilometers, left at least 5 million people without housing, and destroyed millions of livestock and a significant amount of agriculture. The official estimates of the direct economic losses were reported at 123.8 billion USD [14].

In this study, we surveyed adult survivors in the earthquake-affected areas using a multi-stage systematic sampling design. To obtain sufficient power to detect differences in outcomes between the elderly and younger adults, we estimated that 266 participants (133 in each arm) were required to detect a relative risk of 2.5 for PTSD symptoms when the prevalence of the outcome in the comparison group is 10%. Estimates of post-disaster PTSD prevalence in East-Asian populations typically vary between 5% and 30% [5,6,15-17], and we selected the PTSD prevalence (10%) of the 1998 Hunan flood victims in China to calculate the sample size due to the similarity in the impact of the disasters as well as the socioeconomic context [16]. We selected 90% power in the calculation to accommodate the underlying design effect so that 266 participants would provide sufficient power to detect the differences of interest between the two arms. The final sample size was adjusted to 315 subjects (148 elders and 167 younger adults) to account for a potential 10% and 20% non-response rate for the elderly and younger adults, respectively (a higher non-response rate was estimated for the younger adults from pilot data, which could be explained by the internal migration in the area) [18].

**Figure 1:**
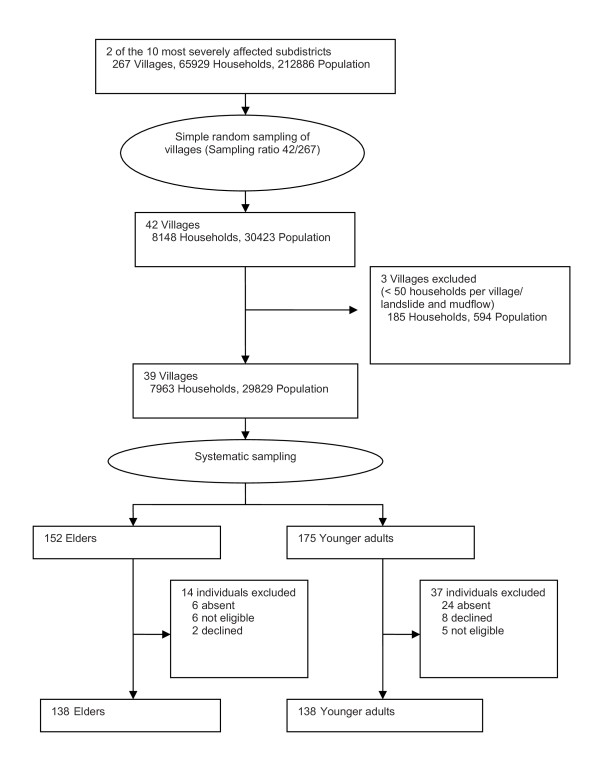
**Sampling strategies for adult survivors of the 2008 Sichuan earthquake, China**.

We randomly selected two of the most severely affected subdistricts from a total of ten [19] (Figure 1). We draw a systematic sample for both elders and younger adults in the available villages with the assistance from the local household registration department. An elder was defined as he or she with at least 60 years of age. When sampling elders, we first calculated a sampling ratio (target number of elders/total number of elders in all of the villages) and then multiplied this ratio by the number of elders in each village [20]. This provided us with the number of elders to be enrolled from each village, proportional to the village size. Next, we calculated a sampling interval (number of elders in the village/number of elders to be sampled from the village), which provided us with the number of people between the two elders to be selected in the sample [20]. At the start of data collection, a randomly generated number between one and ten was used to determine which subject was sampled first; this number was then increased systematically by the sampling interval. Sampling strategies for the younger adults were similar to those of the elderly. As a result, a sample of 152 elders and 175 younger adults was generated, which exceeded the sample size to be required.

### Data Collection

Interviews were carried out by our psychologists (WL, JY, et al.) between August and September, 2009, by which time 15 months had passed since the massive earthquake. A total of 327 adults were targeted. Adults were ineligible if they were not present at the time of the earthquake or had pre-earthquake traumatic experiences (this information was obtained from key informants or the adults themselves, based on which our psychologists judged his or her eligibility). If an adult was not available after two attempts to contact him or her, the adult was omitted and not replaced. A complete description of the survey was presented to the participants before written informed consents were obtained (except the illiterate, who only gave oral consents). The completed questionnaires were carefully examined by the psychologists to ensure that there were no missing values. Adults who were in need of mental health support were referred to local mental health services, and all the visited households were offered a brochure on mental health recovery.

### Instruments

Symptoms of PTSD were measured with the PTSD Checklist-Civilian Version (PCL-C) [21]. The instrument is a self-report 17-item symptom scale that corresponds to DSM-IV criteria [7], and is often used when a clinical interview is not feasible [22,23]. Total score ranges from 17 to 85, and an adult with a score 50 or greater was classified as having probable PTSD. The Internal consistency of the PCL-C in the present study was 0.88.

The General Health Questionnaire (GHQ-12) was used to measure the severity of general psychiatric morbidity (i.e., distress and social dysfunction) of the participants [24,25]. GHQ-12 scores ranged from 0 to 12, with higher scores indicating worse conditions; we used a conventional cut-off for the questionnaire (3/4) to define "cases" of general psychiatric morbidity. The Chinese version of the GHQ-12 used in this study has been validated [26,27]. The Internal consistency of the GHQ-12 in the present study was 0.84.

Earthquake-related experiences were evaluated with an inventory adapted from a prior earthquake exposure scale [28]. Mental health services in the study were defined as interventions conducted by psychologists, psychiatrists, and other certified mental health staff that aimed to promote the mental health recovery of the people surviving the earthquake; people could have received these services in person or in a group, at work or in communities, free or for payment.

The questionnaire was carefully reviewed by our psychologists and linguistic professionals, and was then verified for accuracy and comprehensibility by local mental health staff. The Ethics Committee of the Second Military Medical University approved the procedures and instruments used in the study.

### Statistical Analysis

The PCL-C and GHQ-12 were computed according to the manual. Frequencies, percentages and standard deviations were calculated for descriptive data, *t*-tests were used to evaluate differences in continuous variables, and Chi-square tests were used to test for significance in categorical data. If continuous variables were not normally distributed, Mann-Whitney U tests were applied. Bivariate and backward-conditional multivariate logistic regressions were used to identify independent risk factors for PTSD symptoms and general psychiatric morbidity. All variables with *P *< 0.05 in the bivariate analysis were entered into the multivariate models. The entry and removal criteria for the variables in the multivariate analysis were 0.05 and 0.10, respectively. *P *< 0.05 was considered to be statistically significant. Data were analyzed using SPSS version 17.0 (SPSS Inc, Chicago, Ill).

## Results

Information was gathered from 138 elders and 138 younger adults, and the response rates for the two groups were 90.8% and 78.9%, respectively. Non-response was mostly due to ineligibility or absence. In each group, the respondents and non-respondents did not differ on sex, ethnicity, marital status, or education level. Table 1 shows demographics and earthquake-related experiences of the participants. The Mean (range, SD) age for the elderly and the younger adults was 67.8 (60-82, 5.9) and 33.2 (18-59, 10.3) years (p < 0.01). Compared with the younger adults, the elderly reported higher proportion of being married or living together (81.2% vs. 63.8%; p < 0.01) and of being primarily or lower-educated (65.2% vs. 35.5%; p < 0.01). There were no statistically significant differences between the two groups on the earthquake-related experiences except the loss of family members, as the elders were more likely to report the loss of family members than the younger adults (18.8% vs. 8.7%; p = 0.01).

**Table 1:** Participants characteristics - 2008 Sichuan earthquake, China

		No. (%) of participants			
		
Characteristics		Total (N = 276)	Elders (n = 138)	Younger Adults (n = 138)	*P *Value
Demographics					
Sex					
Male		148 (53.6)	76 (55.1)	72 (52.2)	0.63
Female		128 (46.4)	62 (44.9)	66 (47.8)	
Ethnicity					
Han		110 (39.9)	54 (39.1)	56 (40.6)	0.81
Ethnic minorities ^a^		166 (60.1)	84 (60.9)	82 (59.4)	
Marital status					
Married or living together		200 (72.5)	112 (81.2)	88 (63.8)	0.001
Others (unmarried, divorced, etc.)		76 (27.5)	26 (18.8)	50 (36.2)	
Education level					
Primary school or lower		139 (50.4)	90 (65.2)	49 (35.5)	<0.001
Higher than primary school		137 (49.6)	48 (34.8)	89 (64.5)	
Earthquake-related experiences					
Having been in serious danger					
Yes		167 (60.5)	84 (60.9)	83 (60.1)	0.90
No		109 (39.5)	54 (39.1)	55 (39.9)	
Having been seriously injured					
Yes		33 (12.0)	19 (13.8)	14 (10.1)	0.35
No		243 (88.0)	119 (86.2)	124 (89.9)	
Having family members or friends seriously injured					
Yes		49 (17.8)	26 (18.8)	23 (16.7)	0.64
No		227 (82.2)	112 (81.2)	115 (83.3)	
Having witnessed someone being killed or seriously injured					
Yes		140 (50.7)	70 (50.7)	70 (50.7)	1.00
No		136 (49.3)	68 (49.3)	68 (49.3)	
Having lost family members					
Yes		38 (13.8)	26 (18.8)	12 (8.7)	0.01
No		238 (86.2)	112 (81.2)	126 (91.3)	
Having lost significant others					
Yes		100 (36.2)	49 (35.5)	51 (37.0)	0.80
No		176 (63.8)	89 (64.5)	87 (63.0)	
Having one's house seriously damaged					
Yes		227 (82.2)	117 (84.8)	110 (79.7)	0.27
No		49 (17.8)	21 (15.2)	28 (20.3)	
Having lost important belongings ^b^					
Yes		113 (40.9)	61 (44.2)	52 (37.7)	0.27
No		163 (59.1)	77 (55.8)	86 (62.3)	
Having felt extremely anxious about one's own life					
Yes		226 (81.9)	112 (81.2)	114 (82.6)	0.76
No		50 (18.1)	26 (18.8)	24 (17.4)	
Having felt scared that family members or significant others would die or be seriously injured					
Yes		253 (91.7)	128 (92.8)	125 (90.6)	0.51
No		23 (8.3)	10 (7.2)	13 (9.4)	
Having felt guilt concerning someone's death or injury					
Yes		121 (43.8)	58 (42.0)	63 (45.7)	0.54
No		155 (56.2)	80 (58.0)	75 (54.3)	

^a ^Ethnic minorities refer to non-Han population in China, which account for a larger population in the survey areas than Han people.

^b ^Important belongings refer to physical possessions of sentimental value to the participants.

The point prevalence rates of PTSD symptoms among the elderly and younger adults were 22.5% and 8.0% (p = 0.001), respectively. The point prevalence rates of general psychiatric morbidity among the elderly and younger adults were 42.0% and 25.4% (p = 0.003), respectively. The younger adults reported more utilization of mental health services (19.6% vs. 12.3%), but the difference is not significant (p = 0.10).

Table 2 shows the risk factors for PTSD symptoms among the adult survivors. In the bivariate analysis, a significantly higher prevalence of PTSD symptoms were found among adults who were elderly, reported having been in serious danger, having been seriously injured, having lost family members, having lost significant others, having felt guilt concerning one's death or injury, or having not utilized mental health services. In the multivariate analysis, being elderly (odds ratio (OR) = 3.56, 95% confidence interval (CI): 1.57, 8.06; p = 0.002), having been in serious danger (OR = 3.12, 95% CI: 1.32, 7.37; p = 0.009), having lost family members (OR = 3.43, 95% CI: 1.46, 8.02; p = 0.005), and having felt guilt concerning one's death or injury (OR = 5.22, 95% CI: 2.32, 11.75; p < 0.001) were significantly and independently associated with PTSD symptoms.

**Table 2:** Bivariate and multivariate analysis of PTSD symptoms among the adult survivors

		PTSD, No. (%)	Bivariate OR (95% CI)	*P *Value	Multivariate OR (95% CI)	*P *Value
Overall		42 (15.2)				
Demographics						
Age group						
Younger adults (< 60 y) Elders (≥ 60 y)		11 (8.0) 31 (22.5)	1.00 3.35 (1.61-6.97)	0.001	1.00 3.56 (1.57-8.06)	0.002
Sex						
Male Female		17 (11.5) 25 (19.5)	1.00 1.87 (0.96-3.65)	0.07		
Ethnicity						
Han		17 (15.5)	1.03 (0.53-2.01)			
Ethnic minorities		25 (15.1)	1.00	0.93		
Marital status						
Married or living together Others (unmarried, divorced, etc.)		32 (16.0) 10 (13.2)	1.26 (0.59-2.70) 1.00	0.56		
Education level						
Primary school or lower Higher than primary school		26 (18.7) 16 (11.7)	1.74 (0.89-3.41) 1.00	0.11		
Earthquake-related experiences						
Having been in serious danger						
Yes No		33 (19.8) 9 (8.3)	2.74 (1.25-5.98) 1.00	0.01	3.12 (1.32-7.37) 1.00	0.009
Having been seriously injured						
Yes No		10 (30.3) 32 (13.2)	2.87 (1.25-6.58) 1.00	0.01	NA	
Having family members or friends seriously injured						
Yes No		11 (22.4) 31 (13.7)	1.83 (0.85-3.96) 1.00	0.12		
Having witnessed someone being killed or seriously injured						
Yes		27 (19.3)	1.93 (0.98-3.81)			
Yes No		27 (19.3) 15 (11.0)	1.93 (0.98-3.81) 1.00	0.06		
Having Lost family members						
Yes No		15 (39.5) 27 (11.3)	5.10 (2.37-10.94) 1.00	<0.001	3.43 (1.46-8.02) 1.00	0.005
Having Lost significant others						
Yes No		23 (23.0) 19 (10.8)	2.47 (1.27-4.80) 1.00	0.008	NA	
Having one's house seriously damaged						
Yes 1.00		36 (15.9) 6 (12.2)	1.35 (0.54-3.41) 1.00	0.52		
Having Lost important belongings						
Yes No		21 (18.6) 21 (12.9)	1.54 (0.80-2.98) 1.00	0.20		
Having felt extremely anxious about one's own life						
Yes No		34 (15.0) 8 (16.0)	1.00 1.08 (0.47-2.49)	0.87		
Having felt scared that family members or significant others would die or be seriously injured						
Yes No		40 (15.8) 2 (8.7)	1.97 (0.45-8.74) 1.00	0.37		
Having felt guilt concerning someone's death or injury						
Yes No		31 (25.6) 11 (7.1)	4.51 (2.16-9.42) 1.00	<0.001	5.22 (2.32-11.75) 1.00	<0.001
Utilization of mental health services						
Yes No		1 (2.3) 41 (17.7)	1.00 9.23 (1.24-68.97)	0.03	1.00 7.31 (0.93-57.23)	0.06

Notes: NA (not available), is a marker for variables dropped out of the multivariate model.

Table 3 shows the risk factors for symptoms of general psychiatric morbidity among the adult survivors. In the bivariate analysis, being elderly, having been seriously injured, having family members or friends seriously injured, having witnessed someone being killed or seriously injured, having lost family members, having lost significant others, having lost important belongings, having felt guilt concerning one's death or injury, and having not utilized mental health services were significantly associated with general psychiatric morbidity. In the multivariate analysis, being elderly (OR = 2.14, 95% CI: 1.25, 3.66; p = 0.005), having family members or friends seriously injured (OR = 2.13, 95% CI: 1.10, 4.11; p = 0.03), and having felt guilt concerning one's death or injury (OR = 2.0, 95% CI: 1.18, 3.41; p = 0.01) were significantly and independently associated with general psychiatric morbidity. It is noteworthy that although the utilization of mental health services was not significantly associated with PTSD or general psychiatric morbidity in the multivariate analysis, it was still one of the independent risk factors for both of the symptoms.

**Table 3:** Bivariate and multivariate analysis of general psychiatric morbidity among the adult survivors

		General Psychiatric Morbidity No. (%)	Bivariate OR (95% CI)	*P *Value	Multivariate OR (95% CI)	*P *Value
Overall		93 (33.7)				
Demographics						
Age group						
Younger adults (< 60 y) Elders (≥ 60 y)		35 (25.4) 58 (42.0)	1.00 2.13 (1.28-3.56)	0.004	1.00 2.14 (1.25-3.66)	0.005
Sex						
Male Female		43 (29.1) 50 (39.1)	1.00 1.57 (0.95-2.59)	0.08		
Ethnicity						
Han Ethnic minorities		38 (34.5) 55 (33.1)	1.07 (0.64-1.77) 1.00	0.81		
Marital status						
Married or living together Others (unmarried, divorced, etc.)		72 (36.0) 21 (27.6)	1.47 (0.83-2.63) 1.00	0.19		
Education level						
Primary school or lower Higher than primary school		46 (33.1) 47 (34.3)	1.00 1.06 (0.64-1.74)	0.83		
Earthquake-related experiences						
Having been in serious danger						
Yes No		63 (37.7) 30 (27.5)	1.60 (0.95-2.69) 1.00	0.08		
Having been seriously injured						
Yes No		17 (51.5) 76 (31.3)	2.34 (1.12-4.87) 1.00	0.02	NA	
Having family members or friends seriously injured						
Yes No		25 (51.0) 68 (30.0)	2.44 (1.30-4.56) 1.00	0.005	2.13 (1.10-4.11) 1.00	0.03
Having witnessed someone being killed or seriously injured						
Yes No		57 (40.7) 36 (26.5)	1.91 (1.15-3.17) 1.00	0.01	NA	
Having Lost family members						
Yes No		21 (55.3) 72 (30.3)	2.85 (1.42-5.72) 1.00	0.003	NA	
Having Lost significant others						
Yes No		41 (41.0) 52 (29.5)	1.66 (0.99-2.77) 1.00	0.05	NA	
Having one's house seriously damaged						
Yes No		81 (35.7) 12 (24.5)	1.71 (0.85-3.46) 1.00	0.14		
Having Lost important belongings						
Yes No		47 (41.6) 46 (28.2)	1.81 (1.09-3.01) 1.00	0.02	1.60 (0.94-2.72) 1.00	0.09
Having felt extremely anxious about one's own life						
Yes No		78 (34.5) 15 (30.0)	1.23 (0.63-2.39) 1.00	0.54		
Having felt scared that family members or significant others would die or be seriously injured						
Yes No		89 (35.2) 4 (17.4)	2.58 (0.85-7.81) 1.00	0.09		
Having felt guilt concerning someone's death or injury						
Yes No		52 (43.0) 41 (26.5)	2.10 (1.26-3.48) 1.00	0.004	2.00 (1.18-3.41) 1.00	0.01
Utilization of mental health services						
Yes No		9 (20.5) 84 (36.2)	1.00 .21 (1.01-4.82)	0.05	1.00 2.05 (0.91-4.63)	0.08

Notes: NA(not available) is a marker for variables dropped out of the multivariate model.

## Discussion

In the study, significant distinctions were found in the prevalence of PTSD (22.5% vs. 8.0%) and general psychiatric morbidity (42.0% vs. 25.4%) between elder and younger adult survivors. Arguments may exist on whether the outcome can truly reflect the psychological consequences of the disaster, as it might have been confounded by pre-existing conditions. However, studies show that the PTSD prevalence in the general population of China is well below 1%, and no age difference is reported [29,30]. This adds much confidence to our conclusion that the elderly were more psychologically vulnerable to natural disasters. Nevertheless, the conclusion maybe biased by the limited sample size, case detection technique, and the cross-sectional nature of the study; the imbalanced physical conditions, traumatic exposures, and utilization of mental health services between elder and younger adult survivors may also play a role in interpreting the conclusion. But even after the outcomes were adjusted by a number of confounders using stringent bivariate and multivariate analyses, the post-disaster vulnerability to PTSD and general psychiatric morbidity is still age-dependent.

Studies show that natural disasters may result in feelings of fear, helplessness and vulnerability in many people of all ages [31,32]. The underlying mechanisms that the elderly were more likely to develop psychological problems after disasters are still unclear. But there are some potential reasons. Pekovic et al. argued that due to the fact that an elder often already feels frail because of chronic health conditions, impaired cognitive abilities and decreased sensory awareness, the impact of an unexpected disaster may be overwhelming [3]. Taylor and Vidovic et al. discussed the effect of adrenergic system and the hypothalamic-pituitary-adrenal axis on the neurobiology of PTSD [33,34], although there is no current evidence that the changes seen in these systems with aging affect the development or presentation of post-disaster PTSD in older individuals. Further basic researches are needed to focus on the mechanisms why post-disaster vulnerability to psychological problems is likely to be age-dependent, and provide sound proofs for interventions.

Despite the fact that the work of mental health recovery was critical following the disaster, the road for mental health recovery seems uncertain. Davidson et al. reported that PTSD became chronic in 46% of all patients who developed the disorder [35], and Kessler et al. found that one-third of individuals who developed PTSD after trauma did not have remission of the disorder after ten years [36]. Moreover, past experience in Japan, the Pakistan earthquake, and Hurricane Katrina suggests that chronic medical needs post-disaster are often inadequately managed and can result in increased rates of complications and indirect morbidity after a disaster [37-39]. In our study, we found that only 2.4% of the survivors with PTSD and 9.7% of the survivors with general psychiatric morbidity reported utilization of mental health services, which suggested that a far greater proportion of people with mental health problems had not accessed appropriate care yet. It is also noteworthy that, despite the more crucial situation of psychopathology that elder survivors met, they utilized less mental health services. Additionally, when the elderly were involved in the impaired psychological recovery, the disaster-related impact seems more severe and lasting than we thought [40].

The findings of our study may provide a better understanding of survivors' mental health status after the earthquake. Being elderly, having been in serious danger, having lost family members, having felt guilt concerning one's death or injury, and having not utilized mental health services were independent risk factors for PTSD symptoms, while being elderly, having family members or friends seriously injured, having lost important belongings, having felt guilt concerning one's death or injury, and having not utilized mental health services were independent risk factors for general psychiatric morbidity. These results may help to identify the survivors with an increased risk for either PTSD or psychiatric morbidity. As disaster-related psychological sequelae may last for many years [41,42], actions should be taken to minimize the possible negative impacts. A comprehensive screening program should be established to evaluate the mental health conditions of survivors in earthquake-affected areas at regular intervals, in order to facilitate the early identification of people with mental health problems. Previous studies have also demonstrated the value of community-based mental health aid [43,44]. As the survivors tend not to come for help, outreach services in the community may be effective. It is noteworthy that, the mental health response should not be segregated from the other interventions, because the people not only have psychological needs but also require physical, economic, and spiritual supports as well.

Certain limitations of the study should be recognized. Sichuan earthquake was so powerful and extensive that the shock spread almost half of mainland China. Places had different casualties and losses, and it's hard to select a bunch of people without experiencing the disaster; or when the people were identified as non-exposed, socio-demographics and cultures varied greatly from the exposed. Therefore, it's almost impossible for us to provide comparisons between exposed and non-exposed group within the elder and younger adult survivors. However, we retrieved data from the general population to examine the effect of pre-existing conditions, which added confidence to our conclusions.

## Conclusion

Compared with the younger adults, the elderly survivors were more likely to develop PTSD and general psychiatric morbidity. Despite utilization of mental health services may markedly improve the mental health status, it has been only provided to less than ten percent of the survivors with the mental health symptoms. More mental health services should be distributed to the elderly and groups at particular risk, to ensure their smooth mental health reconstruction after the earthquake.

## Competing interests

The authors declare that they have no competing interests.

## Authors' contributions

ZJ and WT made substantial contributions to conception, design, and interpretation of the data and they involved in drafting the manuscript. All authors participated in the design and performing of the study, acquisition of data and helped to draft the manuscript or revising it. YC performed the statistical analysis. All authors read and approved the final manuscript.

## Pre-publication history

The pre-publication history for this paper can be accessed here:

http://www.biomedcentral.com/1471-2458/10/172/prepub
